# 

**Published:** 1896-02

**Authors:** 


### CONTENTS:

### SELECTIONS.

Article I.—Ancient and Modern Hypnotism. By W. C. Achard. 433
" II.—Cocaine. By J. L. Newborn, D. D. S. - - 444
" III.—Electrolysis for the Surgical Treatment of Strictures. By J. A. Fort, M. D - - - - - - 447
" IV.—Furred Tongue. By Edward C. Kirk, D. D. S. - 449
" V.—The Anesthetic and Asphyxiating Effects of Nitrous-Oxid Inhalation. By J. D. Thomas, D. D. S. - 452
" VI.—Extracting Teeth. By Dr. D. Siddall. - - 461
" VII.—Died Under Gas. - - - - - - - - 464
" VIII.—Dental Enactments. - - - - - - 466

### COMMUNICATED.

Odontographic Society of Chicago. - - - - - - 468

### EDITORIAL, ETC.

Dental Faculties in Medical Schools. - - - - - - 468

### MONTHLY SUMMARY.

Good and Bad Teeth. - - - - - - - 470

Mammoth Teeth Unearthed. - - - - - - - 472

A Case of Complete Separation of the Upper Jaw. - - - - 474

Death Due to Dentistry—A Woman's Fatal Bleeding After Having Sixteen Teeth Pulled. - - - - - - - - 474

Iron and the Teeth. - - - - - - - 475

Circumcision. - - - - - - - - 476

Aluminum Plates. - - - - - - - - 477

Stoned Burs. - - - - - - - - 478

Borine. - - - - - - - - - 478

Character in Teeth. - - - - - - - 479

Beeswax in Canals. - - - - - - - 479

Poisoning by Lysol. - - - - - - - 479

Rules for the Setting of Oxy-Phosphate Cement Fillings. - - 480

A Compatible Antiseptic. - - - - - - - 480

To Lessen the Pain from Arsenic. - - - - - - 480

